# Impact of dietary supplementation of L-citrulline to meat goats during gestation on reproductive performance

**DOI:** 10.1186/s40104-024-01135-z

**Published:** 2025-01-06

**Authors:** Makenzie G. Newton, Arianna N. Lopez, Claire Stenhouse, Karina L. Hissen, Erin D. Connolly, Xingchi Li, Lan Zhou, Guoyao Wu, William B. Foxworth, Fuller W. Bazer

**Affiliations:** 1 Department of Animal Science, Texas A&M University, College Station, Texas, 77843 USA; 2 Department of Veterinary Pathobiology, Texas A&M University, College Station, Texas, 77843 USA; 3 Department of Statistics, Texas A&M University, College Station, Texas, 77843 USA; 4 International Goat Research Center, Prairie View A&M University, Prairie View, Texas, 77446 USA

**Keywords:** Amino acids, Arginine, Citrulline, Early pregnancy, Meat goats

## Abstract

**Background:**

Meat goat production is a worldwide industry with products such as meat, milk, soap, and fiber being produced. There are approximately 2.6 million meat goats in the United States. For breeding female ruminants, early pregnancy loss is estimated to be 30% within the first month of gestation. Extracellular L-citrulline (a precursor to L-arginine) is not degraded by ruminal microbes due to the lack of uptake. L-Arginine and thus L-citrulline, have beneficial impacts on placentation and, subsequently, fetal-placental development and survival. This study aimed to determine the impact of feeding L-citrulline to meat goats during gestation to improve reproductive success. Meat goats were fed either a control (CON) or L-citrulline (CIT) supplemented diet from d 12 to 82 of gestation. Blood samples were collected and sera were subjected to high-performance liquid chromatography analyses to quantify the abundance of amino acids. Pregnancy rates were determined on d 30, 61, and 90 of gestation, and litter weight, individual birth weights, and 90 d adjusted weaning weights were collected.

**Results:**

The concentrations of citrulline, ornithine, and arginine were greater in CIT does compared to CON does, but there was no difference in pregnancy rates between CON and CIT does. Birth weight was greater for male kids born as singles when compared to females, but this phenotype was not observed for kids born as twins or triplets. Further, males born to CON does had greater 90 d adjusted weaning weights than females, but this was not observed in the CIT group. Female kids born to CON and CIT Boer goats had heavier 90 d adjusted weaning weights than those born to Spanish or F1 Boer-Spanish does.

**Conclusion:**

This study provides proof of concept that feeding dietary L-citrulline increases concentrations of citrulline and arginine in blood of gestating meat goats. However, further studies are needed to understand the cellular mechanisms impacted by feeding this supplement. Regardless, this study demonstrated that feeding L-citrulline has the potential to increase reproductive performance in gestating ruminants.

## Background

Meat goats are a popular source of protein in locations such as Asia, Africa, Mexico, Brazil, and the United States [1]. In the United States, there are 2.6 million meat goats with 38% of those animals residing in Texas [2]. Nutritionally, goat meat contains less fat and cholesterol than other animal meats and is an important source of protein and functional amino acids [1]. To ensure continued availability of goat meat for consumption around the world, it is vital that meat goat production continues to increase in efficiency. Unfortunately, early pregnancy loss in ruminant species is a concern, with a large percentage of these losses within the first month of gestation. Thus, there is potential to increase meat goat production if strategies are implemented to mitigate pregnancy loss [3].

L-Citrulline is an amino acid and direct precursor to arginine [4]. Previous studies found that ruminal microbes do not degrade extracellular L-citrulline due to the lack of uptake, meaning that L-citrulline can be fed as an unprotected amino acid and absorbed in the small intestine for conversion into arginine by extrahepatic tissues [5, 6]. Arginine has roles in cell signaling and reproductive processes including embryonic development, implantation, and placentation [7]. Arginine is also required for the production of nitric oxide (NO) and polyamines that directly impact angiogenesis, vasodilation, and the proliferation and migration of cells [4, 7]. Lassala et al. [8] determined that intravenous administration of arginine to ewes from gestational d 100 to 121 enhanced lamb survival and increased birthweights of quadruplets. Further, another study observed that intravenous infusion of arginine from d 60 of gestation until parturition prevented fetal growth restriction in undernourished ewes [9]. It has also been determined that the half-life of citrulline in the blood is longer than that for arginine, suggesting that L-citrulline is potentially a more impactful dietary supplement than arginine because it stays in the circulation longer [10].

The lack of degradation of dietary L-citrulline by ruminal microbes is a potential economic advantage to livestock producers, mainly because no processing of feedstuffs is required to protect this amino acid from ruminal microbial degradation. There is also ease associated with feeding L-citrulline because it can be mixed directly into rations and fed to the livestock. Therefore, the longer half-life and lack of degradation of extracellular L-citrulline in the rumen, in addition to the economic advantage of feeding this amino acid, makes it a potentially favorable supplement to enhance fetal growth and survival in meat goats and other ruminants. We hypothesized that feeding L-citrulline to gestating meat goats would increase concentrations of select amino acids (namely citrulline and arginine) in the maternal circulation to promote fetal development and enhance offspring performance. This hypothesis is based on evidence that dietary supplementation of L-arginine to pregnant pigs between d 30 and 114 of gestation increased the number of live-born piglets by 2 per litter and litter birth-weight by 24% [11]. There is also evidence that dietary supplementation of pregnant rats with arginine increases the number of implantation sites and litter size by approximately three [12, 13]. Additionally, arginine supplementation improves fetal-placental growth and prevents intra-uterine growth restriction of fetuses in pregnant women [14].

## Methods

### Animals

Meat goats (Boer, Spanish, or F1 Boer-Spanish) were group housed at the International Goat Research Center at Prairie View A&M University (Prairie View, Texas, USA; 30.100867, −95.977232) in a covered barn with grazing access and ad libitum access to clean water and feed. On average, the daily intake of feed per goat was 1.4 kg. All experimental procedures followed the Guide for the Use of Agriculture Animals in Research and Teaching and were approved by the Institutional Animal Care and Use Committee at Prairie View A&M University.

### Experimental animals and sample collection

Beginning in September 2022, the estrous cycles of reproductively mature meat goats (*n* = 97) were synchronized by inserting a CIDR (Zoetis, Parsippany, NJ, USA) and administering an injection of GnRH (1 mL Factrel, Zoetis). The does received an injection of prostaglandin (PG) F2α (1 mL Estrumate, Merck, Rahway, NJ, USA) 10 d later. CIDRs were removed 2 d after the PGF2α injection and the does were observed for estrus behavior. The following day, the does were injected with GnRH (1 mL Factrel, Zoetis) and bred via natural service 20 h later. This protocol continued until the last doe was bred in November 2022.

Does were assigned randomly to a breeding group (Groups 1–9) and then separated into treatment groups and fed either a L-citrulline supplemented diet [*n* = 45; 97.63% basal diet + 2% supplement (0.5% L-citrulline, 0.5% L-glutamine as a source of aspartate for arginine synthesis, 1% soybean hydrogenated oil) + 0.37% cornstarch] or an isonitrogenous control diet [*n* = 52; 97.63% basal diet + 1.37% L-alanine + 1% soybean hydrogenated oil] from d 12 to 82 of gestation. Both the isonitrogenous control and treatment diets met the nutritional requirements for gestating goats. The basal diet is listed in Table 1. Whole blood samples were collected from does via jugular venipuncture into vacutainer blood tubes without anticoagulant beginning on d 12 of gestation and for every 14 d until d 82 of gestation. A serial blood draw was performed with one breeding group (*n* = 6 CON, *n* = 6 CIT) of does on d 40 of gestation where whole blood samples were collected immediately before feeding (time 0) and at 30 min, and 1, 2, 4, and 6 h after feeding. Collected samples were stored overnight at 4 °C to clot and centrifuged the following day at 2,600 × *g* for 18 min at 5 °C (Eppendorf 5920R, Hamburg, Germany). Serum was harvested and the samples stored at −20 °C until analyzed to determine concentrations of amino acids in serum of the goats.

**Table 1:** Basal diet for gestating meat goats (as-fed basis)

Ingredient	Content
Protein, %	16.1
Fat, %	3.0
Fiber, %	13.9
Calcium, %	1.1
Phosphorus, %	0.4
Available phosphorus, %	0.13
Total digestible nutrients, %	72.7
Net energy for maintenance, mcal/cwt	77.6
Net energy for gain, %	50.8
Acid detergent fiber, %	15.9
Neutral detergent fiber, %	26.4
Non protein nitrogen, %	1.2
Riboflavin, %	20.0
Salt, %	0.5
Potassium, %	1.1
Sulfur, %	0.2
Magnesium, %	0.2
Manganese, mg/kg	87.5
Iron, mg/kg	173.7
Copper, mg/kg	21.1
Cobalt, mg/kg	0.5
Zinc, mg/kg	71.5
Iodine, mg/kg	0.9
Selenium, mg/kg	0.6
Vitamin A, IU/kg	9,3073.2
Vitamin D, IU/kg	4,409.2
Vitamin E, IU/kg	96.6
Vitamin K, mg/kg	1.1
Thiamine, mg/kg	5.1
Zinc:Copper	3.4
Chlorine, %	0.9
Sodium, %	0.2
Undegradable intake protein, %	37.7
Dry matter, %	89.5
Calcium:Phosphorus	3.0
Decoquinate, g/kg	0.02

Abdominal ultrasonography was performed on d 30, 61, and 90 of gestation to confirm pregnancy. Following kidding, numbers of offspring (including the number of males and females) and birth weights were recorded. Kids were weaned at various ages ranging from 52 to 101 d of age. Therefore, a 90 d adjusted weaning weight was calculated. Adjusted 90 d Weaning Weight was calculated as follows: [(Pre-weaning ADG × 90) + birth weight]. Pre-weaning average daily gain (ADG) was calculated by subtracting birth weight from actual weaning weight and dividing by weaning age [(actual weaning weight – birth weight)/weaning age in days].

### Quantification of amino acids in serum

The abundances of 22 amino acids in serum were quantified using HPLC as described previously [15]. Only serum samples collected during the serial blood draw on d 40 of gestation were analyzed using HPLC to determine systemic changes in concentrations of amino acids in maternal blood relative to time of feeding. Samples (100 µL) were deproteinized using 1.5 mol/L HClO_4_ (100 µL) and 2 mol/L K_2_CO_3_ (50 µL). The mixture was centrifuged at 10,000 × *g* for 3 min. The supernatant was collected and mixed with *o*-phthaldialdehyde (OPA) reagent to perform HPLC analyses using the method involving precolumn derivatization. The OPA reagent was generated by dissolving 50 mg OPA in 1.25 mL HPLC grade methanol (Fisher Scientific, St. Louis, MO, USA). Additionally, 11.2 mL of sodium borate (pH 9.5), 50 µL of 2-mercaptoethnol (M3148 Sigma Aldrich, St. Louis, MO, USA), and 0.5 mL of Brij-23 (B4184 Sigma-Alrich) were added to the mixture. The prepared sample mixture was composed of 1.4 mL of HPLC-grade water (Fisher Scientific), 100 µL of 1.2% benzoic acid (in 40 mmol/L sodium borate, pH 9.5), and 100 µL of sample. In an autosampler (model 712 WISP, Waters, Milford, MA, USA), the assay mixture was derivatized with 30 mmol/L of the OPA reagent, and 15 µL of the mixture was injected into a Supelco 3 μm reversed phase C18 column (150 × 4.6 mm inner diameter, Sigma Alrich). A solvent gradient was generated from solution A (0.1 mol/L sodium acetate, 9% methanol, and 0.5% tetrahydrofuran, pH 7.2) and solution B (HPLC grade methanol) and used to separate amino acids. Amino acids were quantified relative to standards using Millenium 32 Software (Waters).

### Statistical analyses

Pregnancy rates were calculated on d 30, 61, and 90 by the following formula: (number of pregnant does/total number of does in the study) × 100. To check the association between variables, we calculated Pearson’s correlation coefficient for numerical variables and applied chi-square test for categorical variables. A Chi-square value of ≤ 0.05 indicated significance.

A two-sample *t*-test was applied to compare baseline concentrations of amino acids in maternal serum between treatment groups. Linear mixed effects model was used to fit the concentrations of amino acids in maternal serum after Time 0. The final model has concentration at time 0 (baseline), CIT group, Time and Time squared (Time^2^) for fixed effects and random intercept to account for the association within subjects. A significant *P*-value for Time^2^ indicates that the trend observed for changes in concentrations of amino acids over time is quadratic instead of linear.

Standard least squares tests were employed to assess the effect of treatment, group, litter size, breed of doe, fetal sex, and their interactions on overall litter weight, birth weight, and 90 d adjusted weaning weights. Treatments were compared using a least squares means student’s *t*-test. Statistical significance was set at a *P*-value of ≤ 0.05. A 0.05 <*P*-value ≤ 0.10 indicated a trend towards significance. All data analysis was performed using JMP Pro (Version 16.0) and R Statistical Software (Version 4.3.2).

## Results

### Concentrations of amino acids in serum

Concentrations of citrulline, arginine, ornithine, and alanine in maternal serum at different time points after consuming a meal for the CON and CIT groups, as well as *P*-values from one-sided *t*-tests, are summarized in Table 2. Concentrations of the other 18 amino acids are summarized in Table 3. Concentrations of citrulline, arginine, ornithine, and alanine at Time 0 have significant effects on following measurements (*P* < 0.05, Table 2). The concentrations of citrulline (*P* < 0.001) and ornithine (*P* = 0.05) decreased over time in both the CON and CIT treated does, but the CIT does had greater concentrations of these amino acids in serum than CON does (*P* < 0.05; Table 2). Concentrations of arginine in maternal serum decreased numerically for CON does but appeared to be stable in CIT-fed does, as evidenced by the change in concentrations of arginine decreasing less from 0 to 6 h in the CIT group compared to the CON group. Further, there was a tendency for concentrations of arginine to be greater for CIT-fed as compared to CON-fed does (*P* < 0.1; Table 2). The concentrations of alanine were lower in CON does compared to CIT does (*P* < 0.05; Table 2) but remained stable over time in the CON group (*P* > 0.05; Table 2). The interaction between CIT and time has no significant effect (*P* < 0.05, Table 2).

**Table 2:** Concentrations of citrulline, arginine, ornithine, and alanine (nmol/mL) in serum of goats at different timepoints

**Control group**						
**Time**, **h**	**0**	**0.5**	**1**	**2**	**4**	**6**
Citrulline	217.7 ± 41.9	196.4 ± 30.5	184.4 ± 40.6	146.9 ± 30.9	155.1 ± 33.3	152.1 ± 24.1
Arginine	244.8 ± 23.4	217.0 ± 28.8	217.5 ± 29.8	184.3 ± 17.9	212.7 ± 15.3	207.8 ± 17.9
Ornithine	113.3 ± 24.9	90.5 ± 19.2	94.5 ± 21.8	74.1 ± 17.4	88.8 ± 20.5	82.3 ± 14.3
Alanine	180.1 ± 18.0	186.4 ± 21.0	176.6 ± 24.0	155.9 ± 19.4	194.5 ± 16.6	178.7 ± 21.0
**Citrulline group**						
**Time,** **h**	**0**	**0.5**	**1**	**2**	**4**	**6**
Citrulline	217.7 ± 27.2	204.8 ± 23.0	206.3 ± 21.7	184.0 ± 23.7	179.1 ± 18.6	164.6 ± 18.1
Arginine	257.8 ± 15.4	240.2 ± 11.1	241.2 ± 18.5	230.6 ± 22.6	229.8 ± 12.6	231.0 ± 13.2
Ornithine	124.2 ± 12.8	120.9 ± 12.2	120.4 ± 13.1	105.9 ± 12.8	112.1 ± 11.5	108.6 ± 11.6
Alanine	209.7 ± 22.2	229.1 ± 21.5	251.3 ± 23.8	220.5 ± 10.4	227.8 ± 28.2	221.3 ± 30.3
**One-sided two sample** ***t*-test** ***P*-values**						
**Time, h**	**0**	**0.5**	**1**	**2**	**4**	**6**
Citrulline	0.49	0.42	0.32	0.18	0.27	0.34
Arginine	0.32	0.24	0.26	0.07	0.20	0.16
Ornithine	0.35	0.11	0.17	0.09	0.18	0.09
Alanine	0.16	0.09	0.03	0.01	0.17	0.14
***P*-values**						
	**Cit**	**Time**	**Interaction**			
Citrulline	0.020	< 0.001	0.308			
Arginine	0.092	0.359	0.850			
Ornithine	0.001	0.050	0.872			
Alanine	0.046	0.793	0.487			
	**Intercept**^a^	**Concentration at time 0**	**Treatment**	**Time**	**Time**^**2**^	
Citrulline	44.25 ±15.59^*^	0.71 ± 0.06^*^	20.78 ± 9.05^*^	-21.26 ± 6.32^*^	2.18 ± 0.95^*^	
Arginine	43.19 ± 35.73	0.72 ± 0.14^*^	17.35 ± 12.32	-10.42 ± 7.62	1.42 ± 1.15	
Ornithine	10.28 ± 8.35	0.75 ± 0.06^*^	19.38 ± 5.11^*^	-6.73 ± 3.54^*^	0.80 ± 0.53	
Alanine	28.69 ± 29.90	0.86 ± 0.15^*^	26.14 ±14.28^*^	-4.20 ± 7.68	0.52 ± 1.16	

Values represent mean ± SEM. P < 0.05 indicates a significant effect and *P* < 0.1 indicates a trend towards significance

^a^Results from linear effects models, values represent coefficient estimates ± SE

^*^Star indicates a one-sided *P*-value < 0.05

The concentrations of tyrosine, tryptophan, valine, phenylalanine, isoleucine, leucine, lysine and asparagine were typically greater in the CIT-fed does as compared to CON-fed does, except for valine and phenylalanine (*P* < 0.05; Table 3). There was also a tendency for the concentrations of histidine and threonine to be greater in CIT-fed than CON-fed does (*P* < 0.10; Table 3). The concentrations of glycine and taurine decreased over time, while those for beta-alanine increased over time (*P* < 0.05; Table 3). There was also a tendency for threonine to decrease over time in both the CON and CIT does (*P* < 0.10; Table 3).

**Table 3:** Concentrations of amino acids (nmol/mL) in serum of goats at different times after consuming diets supplemented with either alanine (CON) or L-citrulline (CIT)

Treatment	CON						CIT						*P*-value		
Time, h	0	0.5	1	2	4	6	0	0.5	1	2	4	6	Cit	Time	Interaction
Aspartate	16.6 ± 8.7	14.9 ± 5.3	10.0 ± 4.0	9.0 ± 2.6	10.5 ± 5.9	12.8 ± 4.4	14.5 ± 5.8	12.3 ± 4.4	11.2 ± 2.8	16.3 ± 5.3	9.2 ± 1.6	14.4 ± 8.1	0.186	0.647	0.846
Glutamate	125.7 ± 12.1	142.0 ± 17.3	113.1 ± 13.6	130.5 ± 14.5	174.1 ± 47.4	170.1 ± 31.3	121.4 ± 15.0	134.9 ± 19.9	151.2 ± 18.5	173.0 ± 28.9	144.4 ± 21.2	170.1 ± 41.3	0.242	0.172	0.7
Serine	161.4 ± 11.5	156.2 ± 15.6	151.9 ± 19.7	138.9 ± 18.0	147.7 ± 16.3	147.1 ± 15.6	170.3 ± 12.2	169.1 ± 10.5	168.2 ± 13.9	161.8 ± 15.4	168.0 ± 11.4	168.2 ± 13.4	0.182	0.198	0.567
Glutamine	246.1 ± 36.3	247.6 ± 36.1	248.5 ± 43.9	205.3 ± 36.6	240.0 ± 38.0	213.9 ± 40.0	305.7 ± 22.5	325.8 ± 32.7	334.5 ± 25.6	280.6 ± 31.1	351.4 ± 30.8	308.5 ± 34.6	0.107	0.595	0.622
Histidine	64.0 ± 8.1	62.2 ± 7.9	60.6 ± 11.7	51.8 ± 8.3	64.6 ± 8.9	58.2 ± 7.0	72.9 ± 9.7	69.1 ± 6.5	73.7 ± 7.6	67.5 ± 7.7	75.5 ± 8.6	74.4 ± 8.9	0.091	0.733	0.717
Glycine	462.4 ± 61.8	493.6 ± 77.9	442.0 ± 69.9	405.0 ± 65.9	425.9 ± 59.5	421.6 ± 66.0	535.7 ± 36.9	499.7 ± 41.3	501.1 ± 38.0	474.6 ± 32.5	487.6 ± 23.7	471.1 ± 26.6	0.171	0.032	0.339
Threonine	109.6 ± 23.7	111.4 ± 20.0	91.9 ± 18.3	69.8 ± 10.6	96.7 ± 16.7	85.8 ± 12.7	111.8 ± 8.6	114.1 ± 16.7	116.0 ± 15.3	103.6 ± 17.2	106.6 ± 10.9	99.7 ± 10.2	0.095	0.091	0.621
Taurine	138.4 ± 20.1	129.5 ± 17.8	120.0 ± 16.7	104.0 ± 16.9	111.2 ± 16.0	92.5 ± 14.2	141.6 ± 21.3	128.0 ± 19.9	122.3 ± 20.0	109.2 ± 17.6	114.3 ± 18.6	103.2 ± 19.1	0.433	0.000	0.5
Tyrosine	95.7 ± 15.7	88.8 ± 12.1	81.2 ± 13.3	65.8 ± 10.3	85.3 ± 12.0	82.1 ± 10.6	93.4 ± 16.2	96.7 ± 12.2	102.2 ± 13.1	91.6 ± 15.6	102.8 ± 11.5	105.9 ± 13.6	0.006	0.219	0.602
Tryptophan	44.7 ± 1.8	44.6 ± 1.9	40.9 ± 2.0	35.6 ± 3.4	41.2 ± 3.6	37.6 ± 2.7	41.5 ± 7.3	46.0 ± 7.5	48.2 ± 9.0	46.4 ± 8.2	49.1 ± 9.9	47.8 ± 10.5	0.009	0.792	0.261
Methionine	32.9 ± 4.7	28.0 ± 5.2	28.6 ± 5.5	23.9 ± 3.2	28.5 ± 4.7	27.4 ± 3.7	32.1 ± 4.0	28.5 ± 5.5	29.7 ± 4.4	22.9 ± 3.6	29.2 ± 4.5	31.3 ± 6.5	0.359	0.514	0.776
Valine	471.2 ± 110.6	435.9 ± 79.4	405.6 ± 98.5	338.1 ± 67.0	411.9 ± 88.4	355.0 ± 58.9	383.4 ± 43.0	390.5 ± 23.7	410.2 ± 28.1	348.0 ± 30.4	389.8 ± 31.5	380.6 ± 33.5	0.009	0.333	0.717
Phenylalanine	62.3 ± 5.2	58.8 ± 1.9	54.9 ± 4.2	46.8 ± 5.0	62.0 ± 7.5	52.3 ± 4.7	63.0 ± 5.7	59.1 ± 4.6	60.5 ± 2.4	57.8 ± 4.5	64.5 ± 2.7	62.4 ± 4.4	0.018	0.83	0.721
Isoleucine	154.5 ± 28.8	140.1 ± 18.5	127.7 ± 24.5	103.6 ± 15.6	142.4 ± 23.3	123.2 ± 14.3	138.9 ± 11.6	135.2 ± 10.1	140.9 ± 6.3	126.0 ± 12.4	150.6 ± 12.2	146.6 ± 12.8	0.017	0.695	0.626
Leucine	226.7 ± 43.5	203.5 ± 32.3	189.9 ± 38.1	160.9 ± 27.0	200.6 ± 34.5	180.1 ± 17.6	205.6 ± 21.2	189.9 ± 18.2	195.3 ± 15.3	176.6 ± 15.7	205.7 ± 18.2	198.5 ± 22.3	0.004	0.818	0.537
Lysine	203.6 ± 55.8	166.0 ± 41.1	159.4 ± 46.3	112.2 ± 25.3	155.7 ± 32.7	144.9 ± 21.5	198.4 ± 31.0	179.2 ± 22.2	179.6 ± 26.3	157.9 ± 26.9	176.0 ± 19.9	176.5 ± 23.3	0.012	0.285	0.816
Asparagine	54.9 ± 11.0	50.9 ± 12.2	45.6 ± 13.3	29.8 ± 6.2	42.2 ± 10.8	36.2 ± 7.7	62.3 ± 9.4	63.7 ± 7.4	62.7 ± 8.1	45.8 ± 6.9	65.4 ± 7.6	56.4 ± 10.5	0.033	0.198	0.641
Beta-Alanine	19.5 ± 5.1	21.0 ± 5.2	19.5 ± 4.4	20.4 ± 5.1	24.6 ± 5.5	25.2 ± 6.4	14.2 ± 2.0	11.2 ± 2.3	12.7 ± 2.7	12.1 ± 2.8	12.3 ± 3.3	16.4 ± 3.3	0.064	0.001	0.622

Values are means ± SEM. *P* < 0.05 indicates significance; *P* < 0.1 indicates a trend toward significance

### Litter weight, birth weight, and adjusted 90 d weaning weight

The overall pregnancy rates at d 30, 61, and 90 of gestation were 88.5%, 86.5%, and 85.6%, respectively. On d 90 of gestation, the pregnancy rate for CON does was 90.6% and 80.4% for CIT does. Pregnancy rates between CON and CIT-fed does were not statistically different on d 30 or 90 of gestation (*P* > 0.05); however, on d 61 of pregnancy, there tended to be more non-pregnant CIT does than CON does (Prob > ChiSq = 0.072).

**Fig. 1:**
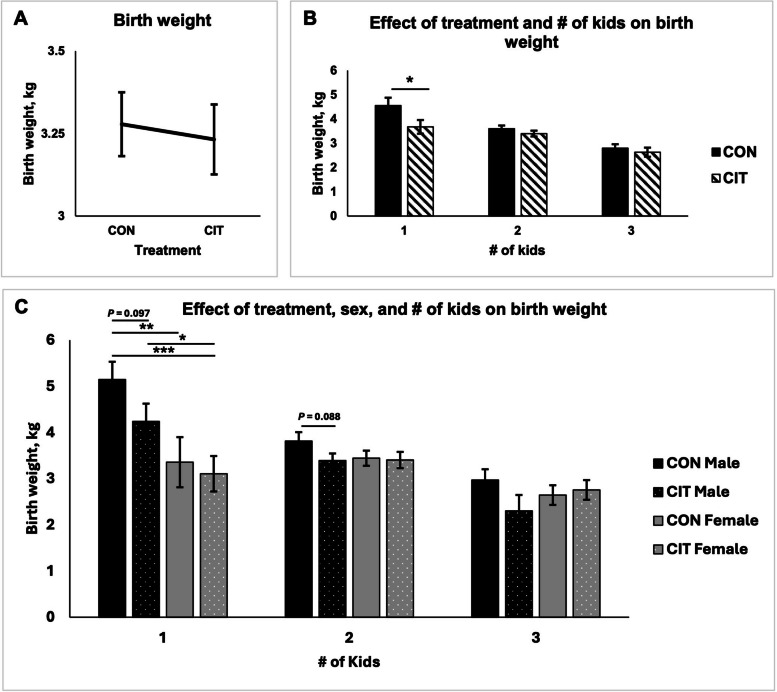
Birth weights of kids born to control (CON) or L-citrulline (CIT) supplemented does. ^*^P < 0.05, ^**^P < 0.01, ^***^P < 0.001. CON refers to the control group and CIT refers to the L-citrulline treated group

Litter weights, obtained by calculating the sum of birth weights after the kids were born, were not affected by CON and CIT diets. Interestingly, there were four does in the CON group that had quadruplets, but none in the CIT group. Dietary L-citrulline supplementation did not affect litter size or birth weights of the kids (Fig. 1A). Single kids born to CON does were heavier than single kids born to CIT does (*P* < 0.05), but there was no treatment effect on birth weights for kids born as twins or triplets (Fig. 1B). Further, when accounting for sex of the offspring and number of kids born per doe, birth weights of single and twin males born to CON does tended to be greater than those born to CIT does (*P* < 0.10), but this phenotype was not observed in males born into a litter of triplets (Fig. 1C). Additionally, birth weights for female offspring were not affected by litter size.

**Fig. 2:**
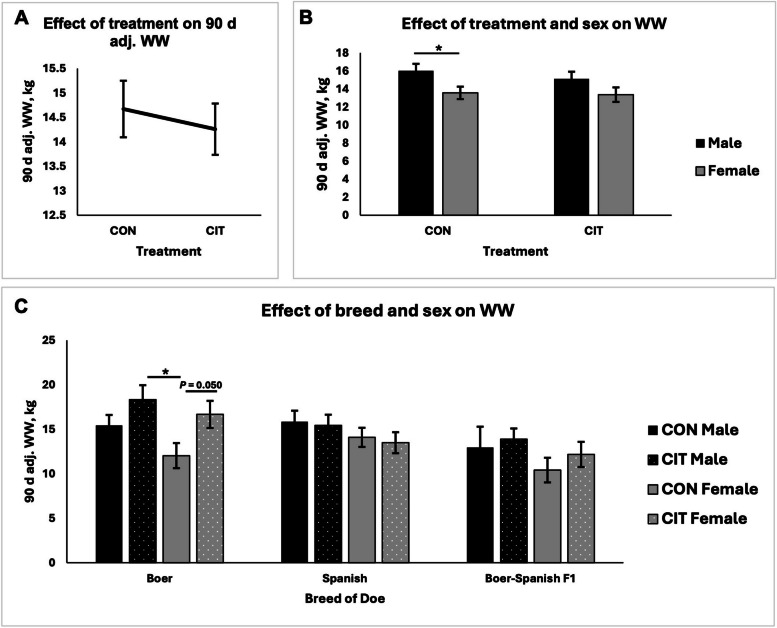
Ninety-day adjusted weaning weights for kids born to control (CON) or L-citrulline (CIT) supplemented does. *WW* Weaning weights. ^*^P < 0.05

There was no difference in 90 d adjusted weaning weights between kids born to CON does and CIT does; however, breed of doe affected 90 d adjusted weaning weights (*P* < 0.05, Fig. 2A and C). When not accounting for breed of doe, but considering effect of sex of the kids, 90 d adjusted weaning weights were greater for male kids born to CON does as compared to female kids born to CON does (*P* < 0.05), but this phenotype was not observed for kids born to CIT does (*P >* 0.05; Fig. 2B). Finally, when accounting for the effect of breed, 90 d adjusted weaning weight was greater for CIT males born to Boer does, and was greater for female kids born to CIT does when compared to the CON kids (*P* = 0.05, Fig. 2C); however, this phenotype was not observed for Spanish or Boer-Spanish F1 does.

## Discussion

Previous studies suggest that L-citrulline has beneficial effects on conceptus growth and survival [4, 16]. This study aimed to determine the systemic concentrations of select amino acids in does fed L-citrulline from d 12 to 82 of gestation and the subsequent performance of their offspring.

Results of the present study indicated that concentrations of citrulline and ornithine (a product of arginine hydrolysis by arginase) were greater in the CIT-fed group but decreased overtime in both the CON and CIT group. Interestingly, concentrations of arginine in maternal plasma decreased over time in CON does, but not in CIT does. The concentrations of arginine tended to be greater in CIT-fed does, suggesting that L-citrulline can stabilize systemic concentrations of arginine in pregnant meat goats. These findings support those from other studies indicating that concentrations of arginine are greater in the plasma of sheep and cattle after consumption of L-citrulline [5, 6]. Further, sheep that consumed rumen protected arginine had greater concentrations of arginine in plasma [17].

The concentrations of alanine, tyrosine, tryptophan, isoleucine, leucine, lysine, and asparagine were lower for CON does than CIT does. More studies are needed to determine the potential roles of these amino acids in gestating meat goats. However, those amino acids are not likely involved in the same metabolic pathways as citrulline and arginine but may be degraded via the pathways that are regulated by arginine and its metabolites. Perhaps supplemental citrulline results in increases in arginine during gestation that contribute more to growth of placental and fetal tissues than to interactions affecting other amino acids.

There was no significant difference in pregnancy rates between CON and CIT groups. Thus, feeding L-citrulline between d 12 and d 82 of gestation neither enhances nor hinders pregnancy outcomes in meat goats and citrulline fed at the current dose had no detrimental effects on gestating meat goats. This finding suggests that L-citrulline is a safe supplement and has the potential to be used in international meat goat enterprises. Previous studies proposed that the presence of 1% soybean hydrogenated oil (originally used to encapsulate citrulline) in the diet may interfere with the action of citrulline, arginine, or its metabolites (such as NO, polyamines, and creatine) in some ruminant species (e.g., sheep) through unknown mechanisms [5, 6, 17]. Therefore, before this supplement could be suggested for utilization in all ruminant operations, studies investigating the absorption, utilization, and metabolic pathways affected by citrulline supplementation should be thoroughly investigated in ruminant species. Furthermore, studies should be conducted to investigate consequences of feeding L-citrulline without the inclusion of 1% soybean hydrogenated oil.

Overall, birth weights were unaffected by treatment; however, single kids born to CIT-fed does were lighter than single kids born to CON-fed does, possibly due to the effect of 1% soybean hydrogenated oil. There was no difference in birth weights among treatment groups for twins and triplets, suggesting that L-citrulline supplementation could be of greater benefit for does gestating multiple fetuses. This observation is consistent with findings of Lassala et al. [8] that intravenous administration of arginine enhanced growth of lambs in ewes with twin or quadruplet pregnancies. This consistent finding suggests that supplementing the diet of ruminant species with L-citrulline during gestation allows for uniformity of fetuses pre-natally and more uniform growth among twins, triplets, and quadruplets, which may contribute to growth rates and, thus, viability of kids post-natally. More research is needed to determine how dietary L-citrulline supplementation to does during gestation impacts energy status, immunity, and overall health of offspring within litters of kids.

The 90 d adjusted weaning weights were greater for males born to CON does when compared to females born to CIT does, but this effect was mitigated in does fed L-citrulline. Further, when the effects of diet were evaluated based on breed of doe, it was observed that for Boer goats, males born to CIT does were heavier than males born to CON does. Similarly, females born to Boer goats in the CIT group were heavier than those in the CON group. The same results were not observed for Spanish or Boer-Spanish F1 does, suggesting that Boer goats are potentially more sensitive to the L-citrulline dietary supplementation than the other breeds of meat goats in this study. This is an interesting finding as the protein and energy requirements for growing Boer and Spanish influenced goats are not known to be different [18, 19]. It has also been suggested that milk from Boer goats is higher in energy content than milk from other breeds of goats; however, further studies are needed to determine if there is a correlation between L-citrulline supplementation, milk yield, and weaning weights of meat goat kids [20].

## Conclusions

Results from this study indicate that feeding unprotected L-citrulline to meat goats from d 12 to 82 of gestation can increase and stabilize circulating levels of arginine in pregnant does. Further, these results suggest that dietary L-citrulline supplementation may be of benefit to does gestating multiple fetuses and that it can increase 90 d adjusted weaning weights for female offspring compared to males. Interestingly, Boer goats appeared to be more sensitive to this supplementation. Collectively, this study revealed that the inclusion of L-citrulline in diets of pregnant meat goats may be beneficial for improving fetal growth and overall reproductive performance. A major limitation of this study was the need to group-feed does on the respective treatments. Therefore, the exact intake of dietary L-citrulline could not be determined. Future studies of meat goats fed individually are needed for dose-response studies to better define optimal rates of the dietary supplementation of L-citrulline. Additionally, the underlying mechanisms involved in and impacted by L-citrulline supplementation and its impact on reproductive performance of meat goats, other species of livestock, and humans should be investigated.

The practical aspect of feeding L-citrulline to improve reproductive performance of ruminant species is advantageous for livestock producers, due to the ease of exposing gestating animals to the supplement. L-citrulline has the potential to enhance pregnancy outcomes by stabilizing circulating concentrations of arginine in gestating animals. An increased understanding of how nutrition impacts pregnancy and fetal development is vital for successful livestock production and the continual supply of animal products for use in daily life.

## Data Availability

The datasets presented in this paper can be made available by the corresponding author upon request.

## Abbreviations

ADG: Average daily gain
CIT: Citrulline-fed group
CON: Control-fed group
HPLC: High performance liquid chromatography
NO: Nitric oxide
OPA: *o*-Phthaldialdehyde
